# LncRNA RP11-297P16.4 Promotes the Invasion and Metastasis of Non-Small-Cell Lung Carcinoma by Targeting the miR-145-5p/MMP-2/9 Axis

**DOI:** 10.3390/biomedicines13030617

**Published:** 2025-03-03

**Authors:** Wei Wang, Yu Lu, Guang-Mei Qin, Lin-Feng Ni, Bai-Xue Xu, Chao-Feng Liu, Bao-Feng Yu, Hai-Long Wang, Min Pang

**Affiliations:** 1 School of Basic Medical Sciences, Basic Medical Science Center, Institute of Cancer Biology, Shanxi Medical University, Jinzhong 030600, China; wwsxmed@163.com (W.W.); 17505954961@163.com (Y.L.); 18103272748@163.com (G.-M.Q.); jonny2lee00@gmail.com (L.-F.N.); xbx76908973@163.com (B.-X.X.); liucfty@163.com (C.-F.L.); shanxiyangcheng@126.com (B.-F.Y.); 2 NHC Key Laboratory of Pneumoconiosis, Shanxi Province Key Laboratory of Respiratory Disease, Department of Pulmonary and Critical Care Medicine, The First Hospital, Shanxi Medical University, Taiyuan 030001, China

**Keywords:** lncRNA RP11-297P16.4, non-small-cell lung carcinoma, miR-145-5p, matrix metalloproteinase, invasion, migration

## Abstract

**Background/Objectives:** Long noncoding RNAs (lncRNAs) participate in the occurrence and development of non-small-cell lung carcinoma (NSCLC). But for certain lncRNAs, their effects on NSCLC remain unclear. This work discovered that lncRNA RP11-297P16.4 is elevated in NSCLC. **Methods:** LncRNA RP11-297P16.4 expression within LUAD tissues and cells was measured through RT-qPCR and Western blot. To assess the role of the lncRNA RP11-297P16.4 in NSCLC, gain- or loss-of-function experiments were conducted using an NSCLC mouse tumor model. **Results:** Silencing of the lncRNA RP11-297P16.4 inhibited the NSCLC cell line invasion and migration potential, but re-expression of the lncRNA RP11-297P16.4 had the opposite effect. A luciferase reporter confirmed that the lncRNA RP11-297P16.4 functions as a competitive endogenous RNA (ceRNA) through the sponge of miR-145-5p. The expression of lncRNA RP11-297P16.4 was negatively correlated to the level of miR-145-5p in NSCLC cells, which sponged miR-145-5p and suppressed tumor cell migration and invasion by targeting matrix metalloproteinase 2 (MMP-2) and MMP-9. **Conclusions:** Our findings suggested that the lncRNA RP11-297P16.4/miR-145-5p/MMP-2/9 regulatory axis is the key pathway for mediating the migration and invasion of NSCLC.

## 1. Introduction

Lung cancer is the most common cancer in the world, accounting for 12.4% of all new cases and 18.7% of all cancer-related deaths, making it the leading cause of cancer-related death in the world [1]. Lung cancer is divided into two main subtypes according to its histology: small-cell lung carcinoma (SCLC), which accounts for 15% of all cases, and non- small-cell lung carcinoma (NSCLC), which accounts for 85% of all cases. NSCLCs have three histologic types: squamous-cell carcinoma, adenocarcinoma, and large-cell carcinoma [2]. Lung adenocarcinoma (LUAD), which develops from small airway epithelial type II alveolar cells, is the most common type of lung cancer and accounts for about 35~40% of all cases of lung cancer [3,4]. Large-cell carcinomas constitute approximately 10% of NSCLC cases [5]. Significant progress has been made in diagnostic methods, targeted drugs, and immunotherapy, but overall survival (OS) for LUAD patients remains unsatisfactory, with an average five-year survival rate of less than 20% due to cancer cell diffusion and metastases [6,7]. However, the mechanisms underlying the occurrence of NSCLC are not clear. Thus, identifying cancer-related biomarkers might enhance the early detection of NSCLC.

Long noncoding RNAs (lncRNAs), which are related to the etiology of NSCLC, play important roles in regulating gene expression [8,9,10]. For example, the lncRNA ZBED5-AS1 facilitates tumor progression and metastasis in LUAD via the ZNF146/ATR/Chk1 axis [11]. The lncRNA XIST inhibits ferroptosis by regulating GPX4 to promote the growth of LUAD, which is closely associated with poor prognosis [12]. Moreover, lncRNAs are endogenous RNAs (ceRNAs) that competitively bind to microRNAs (miRNAs) and reduce their regulatory effect on target mRNAs [13]. LINC01554 could regulate NSCLC cells proliferation, migration, invasion, and epithelial–mesenchymal transition (EMT) via functioning as a ceRNA for miR-1267 [14]. The lncRNA UCC promotes EMT via the miR-143-3p/SOX5 axis in NSCLC [15]. EMT is a critical process in cancer progression, enabling tumor cells to acquire migratory and invasive properties that drive metastasis and therapeutic resistance [16]. Several investigative studies have found that tumor-associated matrix metalloproteinases (MMPs), like MMP-2 and MMP-9, can also stimulate the EMT or its related processes [17,18].

A previous study showed that lncRNA RP11-297P16.4 was elevated in gastric cancer, indicating it was involved in the occurrence and development of tumors, while its functions remained unknown [19]. Furthermore, the expression and function of lncRNA RP11-297P16.4 in NSCLC also remained unclear. Therefore, the Cancer Genome Atlas (TCGA) project and GEPIA (Gene Expression Profiling Interactive Analysis) were used to analyze the expression of lncRNA RP11-297P16.4 in LUAD. Next, the levels of the lncRNA RP11-297P16.4 in tumor tissues from LUAD patients were assayed, and the function of the lncRNA RP11-297P16.4 in NSCLC cell lines was evaluated *in vitro* and *in vivo*. Our findings revealed that the lncRNA RP11-297P16.4 was overexpressed in tumor tissues and that it participated in the invasion and migration of NSCLC cell lines. Moreover, lncRNA RP11-297P16.4 was found to affect NSCLC cell lines by sponging miR-145-5p to modulate MMP-2 and MMP-9, which might stimulate processes associated with EMT.

## 2. Materials and Methods

### 2.1. Cell Culture

Normal bronchial epithelial cells (HBECs), a LUAD cell line (A549 cells), and a large-cell carcinoma cell line (H1299 cells) were acquired from the Cell Culture Center of the Chinese Academy of Medical Sciences (Beijing, China). A549 cells were cultivated in McCoy’s 5A medium (Boster Biological Technology, Pleasanton, CA, USA) supplemented with 10% fetal bovine serum (FBS), whereas H1299 cells were cultivated in 10% FBS-containing RPMI-1640 medium (Boster Biological Technology). The precursor sequence of lncRNA RP11-297P16.4 was cloned and inserted into the lentivirus backbone plasmid LV10N (U6/mCherry&Puro; Shanghai GenePharma Co., Ltd., Shanghai, China). The A549 and H1299 cell lines were subjected to infection with packaged lentivirus for one week using 2.5 mg/mL puromycin. Subsequently, stable cell lines with downregulated RP11-297P16.4 expression marked as short hairpin RNA-RP11-297P16.4 (sh-RP11-297P16.4) were established. The efficacy of RP11-297P16.4 knockdown was determined through reverse-transcription quantitative PCR (RT-qPCR).

### 2.2. Transient Transfection

RP11-297P16.4 was overexpressed through the pLenti-CMV-RP11-297P16.4-GFP-Puro plasmid (OE-RP11-297P16.4), with the Plenti-CMV-GFP-Puro plasmid used as the control (OE negative control, OE-NC). These plasmids were obtained from the Public Protein/Plasmid Library (Nanjing, China). Additionally, miR-145-5p mimics, inhibitors, and relevant control groups were obtained from Shanghai Genepharma, Inc (Shanghai, China). Lipofectamine 2000 (Invitrogen, Waltham, MA, USA) was used for plasmid transfection following specific protocols.

### 2.3. CCK-8 Assay

A Cell Counting Kit-8 (CCK-8) provided by Boster Biological Technology (AR1160) was used to detect cell proliferation. We inoculated cells (2 × 10^3^/well) in 96-well plates. The CCK-8 kit solution (10 μL) was added to each well, after which the cells were incubated at 37 °C for 2 h. A microplate reader (SpectraMax^®^, 190, San Jose, CA, USA) was used to measure the absorbance (OD) value of the cell culture supernatants at 450 nm. All experiments were conducted in triplicate.

### 2.4. Colony Formation Assay

Briefly, cells were inoculated in six-well plates at a density of 1000 cells/well in triplicate, and the cells were cultured for 10 days. Then, cells were fixed with 4% paraformaldehyde for 20 min at room temperature, followed by PBS washing. Fixed colonies were stained with 0.1% crystal violet for 10 min, rinsed thoroughly with PBS, and air-dried. The number of colonies was manually calculated in the treated and control cells. Colonies with more than 50 cells were counted.

### 2.5. Wound Healing Assay

First, H1299 and A549 cells were cultivated in a six-well plate. Upon reaching 90% confluence, the cells were washed with phosphate-buffered saline (PBS), and linear wounds were made using a sterile 1000-μL pipette tip. Then, the cells were cultured for 48 h in a culture medium supplemented with 1% FBS. An optical microscope was used to observe the extent of the wound, and photos were taken. Each assay was conducted three times.

### 2.6. Transwell Assay

We inoculated 5000 cells in the top transwell chamber (8.0 μm, Costar, Corning, NY, USA) containing serum-free medium (300 μL) and introduced 10% FBS-containing medium (600 μL) in the bottom chamber. For cell invasive ability, we coated Matrigel (BD Biosciences, San Jose, CA, USA) onto the top chamber before the experiment and used it to reconstitute the basement membrane, occluding the membrane pores while blocking noninvasive cell migration via the membrane. Following 48 h of incubation, a cotton swab was used to eliminate cells in the top chamber, whereas invading cells at the bottom of the membrane were fixed and stained with 0.1% crystal violet. Subsequently, five fields were selected at random to calculate the number of invading cells. To evaluate migration, no Matrigel was used, whereas the other assay steps were the same.

### 2.7. RNA Isolation and RT-qPCR Assay

An RNA prep pure tissue kit (LS1040, Promega, Madison, WI, USA) was used to extract total cellular RNA following specific protocols. The RT—PCR Kit (Perfect Real Time) (MF166-01, Mei5 Biotech, Beijing, China) was used to prepare cDNA. A StepOnePlus™ Real-Time PCR System (Applied Biosystems, Thermo Fisher Scientific, Waltham, MA, USA) was used for RT-qPCR with SYBR 2× M5 HiPer Real-time PCR reagents (MF797-01, Mei5 Biotech, China). The primers used for RT-qPCR were as follows: lncRNARP11-297P16.4-F: CCCGTCCTCCTACACTTTG and lncRNARP11-297P16.4-R: CCCGTCCTCCTACACTTTG. β-actin was used as the endogenous reference for RT-qPCR normalization. The comparative Ct approach (2^−ΔΔCt^) was applied to determine the gene level.

### 2.8. Western Blot Assay

SDS lysis buffer (10% glycerol, 2% SDS, and 50 mM Tris-HCl/pH 6.8) was added to extract total proteins, followed by separation through a 10% SDS—PAGE gel and transfer to polyvinylidene difluoride (PVDF) membranes. After being blocked with 5% nonfat milk, the membranes were probed with rabbit monoclonal or polyclonal antibodies against MMP-2 (bs-4599R), MMP-9 (bs-4593R, Bioss Biotechnology, Beijing, China), β-actin (Santa Cruz, Dallas, TX, USA), and GAPDH (BioWorld, Irving, TX, USA). Then, the membranes were washed and incubated with HRP-labeled goat anti-rabbit (BA1054) and anti-mouse (BA1050) secondary antibodies (1:5000, Boster Biological Technology, China) for 1 h, followed by analysis using an enhanced chemiluminescence (ECL) Western blot analysis system (ProteinSimple, AlphalMager, MINI, San Jose, CA, USA). The protein band gray value density was quantified using ImageJ software 1.8.0.

### 2.9. Cell Cytoplasm/Nucleus Fraction Isolation

Cytoplasmic/nuclear cellular RNAs were separated and purified using an RNA Subcellular Isolation Kit (Active Motif, Carlsbad, CA, USA). An RT-qPCR assay was subsequently conducted on cytoplasmic/nuclear RNAs to detect nuclear/cytoplasmic control transcript (U6/GAPDH separately) and RP11-297P16.4 expression.

### 2.10. Dual-Luciferase Reporter Assay

A549, H1299, and 293T cells (2 × 10^4^/well) were inoculated in 24-well plates overnight. On day 2, the cells were co-transfected with the pmirGLO-RP11-297P16.4-wild type (WT) or pmirGLO-RP11-297P16.4-mutant (MUT) reporter plasmid and the miR-145-5p mimic or inhibitor. At 24 h following transfection, a dual-fluorophore enzyme reporter gene assay kit (Biyuntian Biotechnology Co., Shanghai, China) was used to measure luciferase activity relative to Renilla luciferase activity.

### 2.11. Xenograft Assay

All animal experiments were approved by the Animal Ethics Committee of Shanxi Medical University (approval number SYDL-2020016), and the experiments were performed strictly following the ARRIVE guidelines. We purchased 4–6-week-old female BALB/c nude mice (*n* = 10) from Beijing HFK Bioscience Co., Ltd. (Beijing, China). The animals were housed in a specific pathogen-free environment (23 °C, 60% relative humidity, 12 h/12 h light–dark cycle, and ad libitum water and food). The mice were placed in one of two groups (*n* = 5 mice/group). Each mouse was subsequently given a subcutaneous injection of short hairpin (sh) RNA negative control (sh-NC)/A549 or sh-RNA RP11-297P16.4/A549 (4 × 10^6^ cells/mouse) via the lateral tail vein. The tumor growth and health of each mouse were monitored daily. Four weeks later, the animals were intraperitoneally injected with sodium pentobarbital (75 mg/kg body weight) for anesthesia before their lung tissue was collected to count the number of metastases on the surfaces. The tissues underwent 4% paraformaldehyde fixation before hematoxylin and eosin staining or immunohistochemical staining. For immunohistochemical analysis, the slices were blocked with bovine serum albumin (BSA). After removing the mixture, primary antibodies against MMP-2 (1:100), MMP-9 (1:100), NKX2-1 (1:100, Sino Biological, Beijing, China), and Napsin A (1:100, Boster Biological Technology) were added, and the slices were incubated with secondary antibodies for 1 h, followed by DAB coloring and restaining with hematoxylin. After dehydration and transparency, the slices were sealed for observation.

### 2.12. Clinical Specimens

We downloaded RNA-seq data of NSCLC and lncRNA-RP11-297P16.4 and the miR-145-5p expression profiles from the TCGA dataset. All tissue cDNAs were provided by our center [20], and the protocols were approved by the ethics committee of the First Hospital of Shanxi Medical University (approval number 2019K042).

### 2.13. Statistical Analysis

All data were analyzed using GraphPad Prism 9.5 and SPSS 20.0, with at least three independent replicate trials conducted for each experiment. The differences in parameters between groups were determined by an independent-samples *t*-test, and *p* < 0.05 indicated a significant difference.

## 3. Results

### 3.1. LncRNA RP11-297P16.4 Is Upregulated in LUAD Tissues

**Figure 1:**
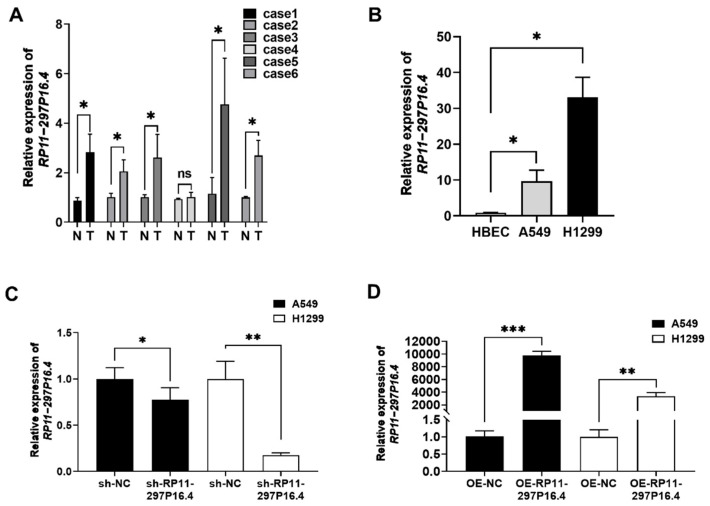
RT-qPCR analysis of lncRNA RP11-297P16.4 expression in LUAD tumor tissues and NSCLC cell lines. (**A**) RP11-297P16.4 levels in six paired human LUAD and matched non-carcinoma samples were examined through RT-qPCR assays (N: normal tissues; T: tumorous tissues; ns: not significant). (**B**) RP11-297P16.4 expression in NSCLC cells and HBECs was analyzed by RT-qPCR. (**C**) RT-qPCR assay of lncRNA RP11-297P16.4 knockdown in A549 and H1299 cells. (**D**) RT-qPCR assay of the overexpression of lncRNA RP11-297P16.4 in A549 and H1299 cells. Data are presented as mean ± SD; * *p* < 0.05, ** *p* < 0.01, and *** *p* < 0.001.

RT-qPCR was performed to characterize the lncRNA RP11-297P16.4 in LUAD tissues and showed that LUAD tissues presented higher lncRNA RP11-297P16.4 expression than matched non-carcinoma lung tissue samples (Figure 1A). Additionally, lncRNA RP11-297P16.4 expression was substantially higher in NSCLC cell lines (A549 and H1299 cells) than in HBECs (Figure 1B).

### 3.2. LncRNA RP11-297P16.4 Enhances A549 and H1299 Cell Invasion and Migration

To analyze the effect of RP11-297P16.4 on NSCLC development, RP11-297P16.4 was knocked down by LV10N (U6/mCherry&Puro) lentivirus and overexpressed using the plenti-cmv-GFP-puro vector in A549 and H1299 cells.

**Figure 2:**
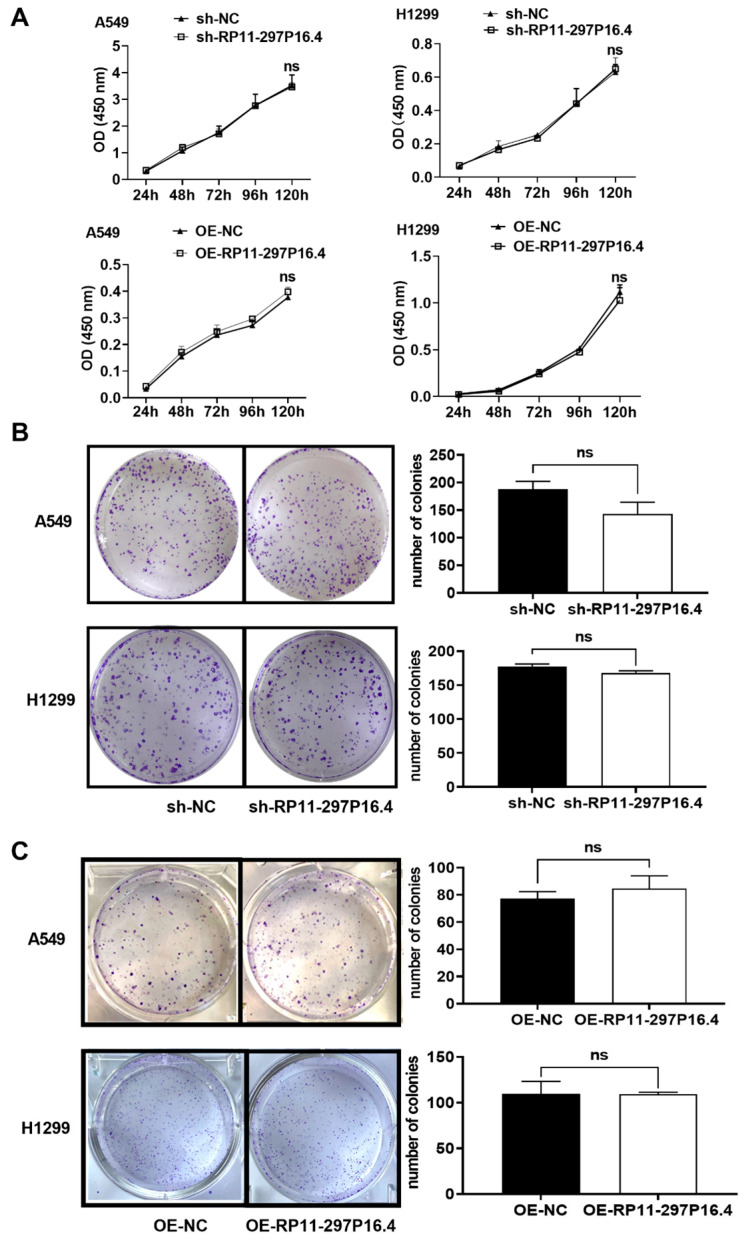
CCK-8 and colony formation assays were performed to determine the effects of knocking down and overexpressing lncRNA RP11-297P16.4 on the growth of A549 and H1299 cells. (**A**) CCK-8 assay of growth ability following the transfection of sh-RP11-297P16.4 (RP11-297P16.4-knockdown) or OE-RP11-297P16.4 (RP11-297P16.4-overexpressing) in A549 and H1299 cells. (**B**) Colony formation assay of the growth ability of these cells following sh-RP11-297P16.4 transfection. (**C**) Colony formation assay of the growth ability of A549 and H1299 cells following OE-RP11-297P16.4 transfection. Data are presented as mean ± SD. Abbreviations: ns, no significant difference vs. NC group.

We conducted RT-qPCR assays to determine whether RP11-297P16.4 expression was modulated in both cell lines (Figure 1C,D). To assess the role of RP11-297P16.4 in cell growth, we conducted CCK-8 and colony formation assays. The results showed that the decrease or increase in the level of the lncRNA RP11-297P16.4 did not affect cell proliferation (Figure 2).

**Figure 3:**
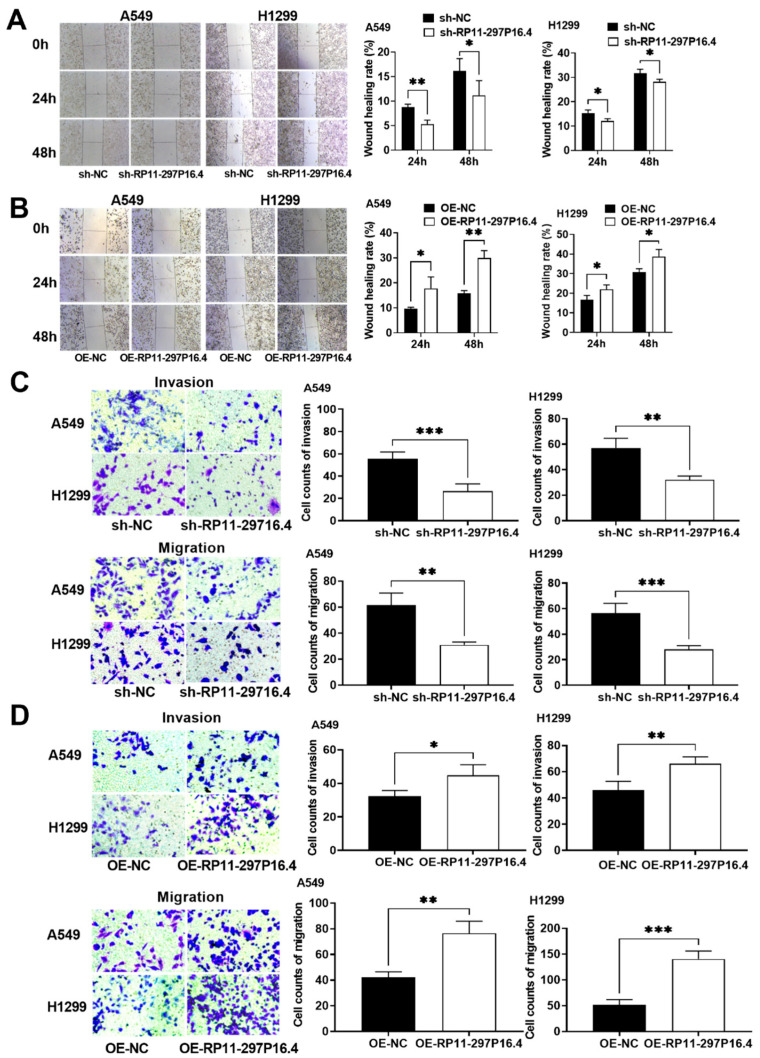
Wound healing and transwell assays were conducted to determine metastasis in A549 and H1299 cells in which lncRNA RP11-297P16.4 was knocked down or overexpressed. Wound healing assays were conducted to determine the metastatic ability following sh-RP11-297P16.4 transfection (**A**) and OE-RP11-297P16.4 transfection (**B**) in A549 and H1299 cells. Transwell assays were performed to determine the invasion and migration abilities following sh-RP11-297P16.4 transfection (**C**) and OE-RP11-297P16.4 transfection (**D**) in A549 and H1299 cells. Data are presented as mean ± SD; * *p* < 0.05, ** *p* < 0.01, and *** *p* < 0.001.

We also conducted wound healing and transwell assays to assess the function of the lncRNA RP11-297P16.4 in cellular metastasis. It was found that the overexpression of the lncRNA RP11-297P16.4 (RP11-297P16.4 OE) increased the metastasis of both cell lines. In contrast, lncRNA RP11-297P16.4 silencing considerably reduced the metastasis of both cell lines (Figure 3).

In LUAD, especially metastatic tumor cells, the increased expression of MMP-2 and MMP-9 levels is related to basement membrane (BM) and extracellular matrix (ECM) degradation [21]. They also promote metastasis by helping cancer cells detach from the primary site. Therefore, we investigated whether the lncRNA RP11-297P16.4 facilitates NSCLC cell invasion and migration by modulating the MMP-2 and MMP-9 levels.

**Figure 4:**
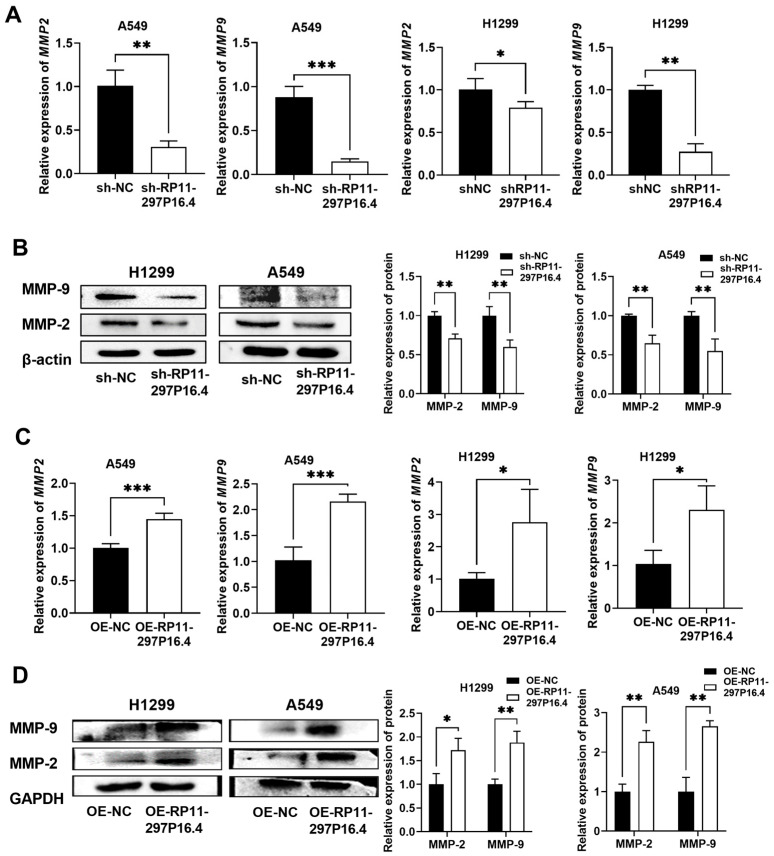
LncRNA RP11-297P16.4 increased MMP-2 and MMP-9 levels in NSCLC cells. A: RT-qPCR assays of sh-RP11-297P16.4-knockdown (**A**) and lncRNA RP11-297P16.4-overexpressing (**C**) in A549 and H1299 cells. Western blot analysis of MMP-2 and MMP-9 levels following sh-RP11-297P16.4 transfection (**B**) and lncRNA RP11-297P16.4 plasmid transfection (**D**) in A549 and H1299 cells. Data are presented as mean ± SD; * *p* < 0.05, ** *p* < 0.01, and *** *p* < 0.001.

The levels of MMP-2 and MMP-9 were detected by RT-qPCR and Western blot (WB) assays in A549 and H1299 cells with lncRNA RP11-297P16.4 silencing or overexpression. The expression of lncRNA RP11-297P16.4 was positively related to MMP-2/9, suggesting that lncRNA RP11-297P16.4 might positively regulate the post-transcriptional expression of MMP-2 and MMP-9 in NSCLC cells *in vitro* (Figure 4).

### 3.3. LncRNA RP11-297P16.4 Promotes NSCLC Cell Invasion and Migration In Vivo

**Figure 5:**
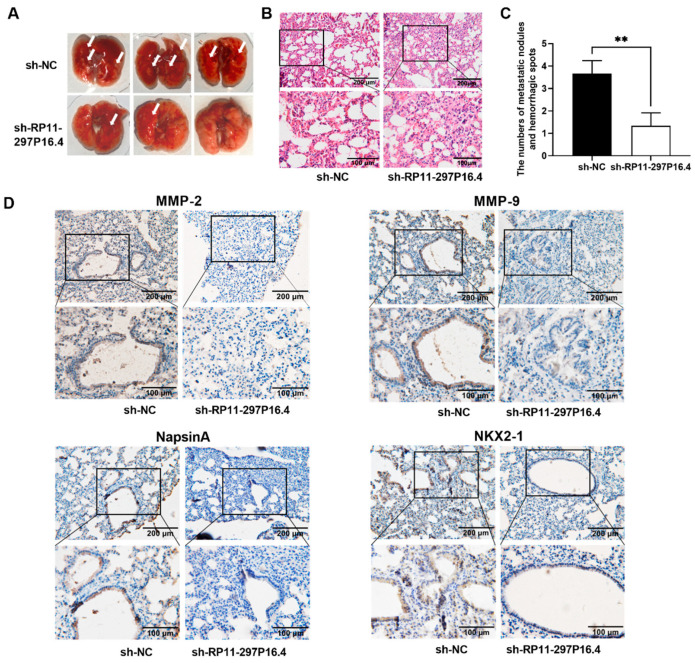
Knockdown of lncRNA RP11-297P16.4 suppressed xenograft tumor metastasis. (**A**) Representative images showing hemorrhagic spots and metastatic nodules (white arrows) in mouse lungs inoculated with sh-RP11-297P16.4- or sh-NC-transfected A549 cells. (**B**) H&E staining for morphological alterations in the tumor metastasis of the sh-RP11-297P16.4 and sh-NC groups. (**C**) Tumor numbers and hemorrhagic spots on the mouse lung surface were compared between the sh-RP11-297P16.4 and sh-NC groups. (**D**) NKX2-1, Napsin A, MMP-2, and MMP-9 immunoreactivities in mouse lung tissues from the sh-RP11-297P16.4 and sh-NC groups. Data are presented as mean ± SD; ** *p* < 0.01.

To elucidate the effect of lncRNA RP11-297P16.4 on cancer invasion and migration *in vivo*, we injected sh-RP11-297P16.4 or its sh-NC A549 cells into nude mice via their lateral tail vein. At 28 days post-implantation, the number of hemorrhagic spots and metastatic nodules was considerably lower in the lungs of sh-RP11-297P16.4 A549 cells compared to that in the lungs of their sh-NC A549 cell-injected counterparts (Figure 5A). Hematoxylin and eosin (H&E) staining of lung sections showed that the cells in the sh-NC A549 cell injection group were irregularly arranged, with most cells surrounding blood vessels, and a large number of erythrocytes was present. In contrast, the sh-RP11-297P16.4-A549 cell group presented significantly fewer cells in the same areas of mouse lung tissue (Figure 5B). The number of tumors was also lower in the sh-RP11-297P16.4-A549 cell group (Figure 5C). Immunohistochemical assays targeting MMP-2, MMP-9, and the LUAD cell markers NKX2-1 [22] and Napsin A [23] in mouse lung tissues showed that, compared to the sh-NC A549 cell-injected nude mice, the sh-RP11-297P16.4-A549-injected mice presented fewer MMP-2- and MMP-9-positive particles. Additionally, NKX2-1-positive and Napsin A-positive cells were more abundant in the sh-NC A549 injection group than in the sh-RP11-297P16.4-A549 injection group (Figure 5D), suggesting that sh-RP11-297P16.4 reduced the protein expression levels of NKX2-1 and Napsin A in mouse lung tissues. All of these results indicated that lncRNA RP11-297P16.4 enhances A549 cell invasion and migration *in vivo*.

### 3.4. LncRNA RP11-297P16.4 Is the ceRNA That Binds to miR-145-5p

**Figure 6:**
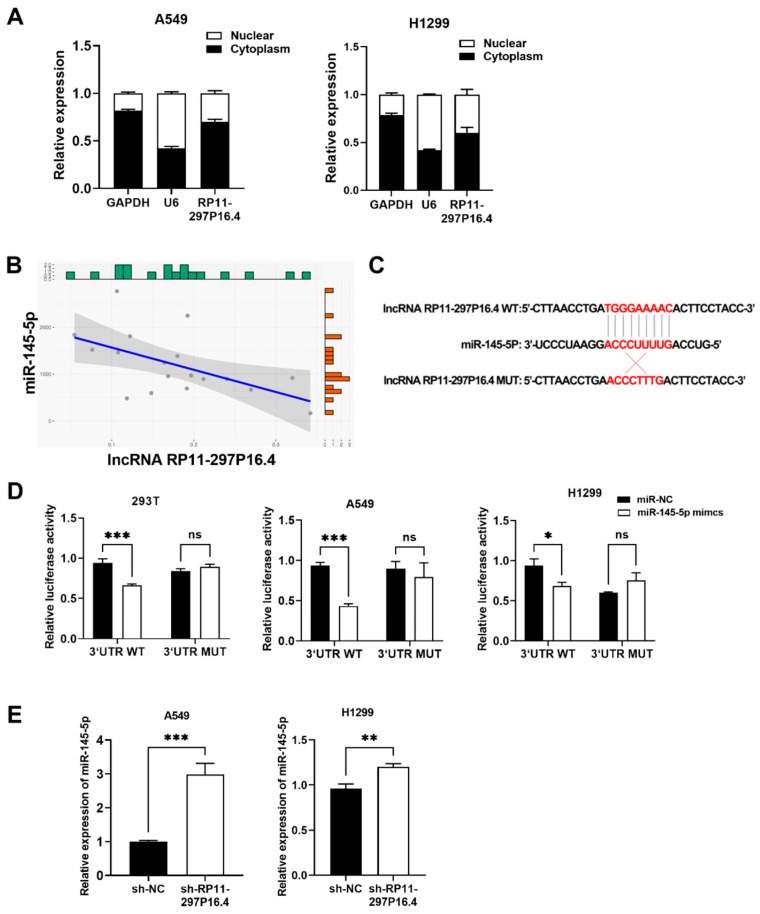
LncRNA RP11-297P16.4 bound to miR-145-5p. (**A**) A subcellular localization experiment was performed to show the localization of the lncRNA RP11-297P16.4 in cells. RT-qPCR assay was subsequently conducted on cytoplasmic/nuclear RNAs to detect nuclear/cytoplasmic control transcript (U6/GAPDH separately) and RP11-297P16.4 expression. (**B**) Pearson analysis of the relationship between lncRNA RP11-297P16.4 and miR-145-5p expression in LUAD samples. (**C**) A map illustrating the miR-145-5p binding site with WT or MUT lncRNA RP11-297P16.4. (**D**) Luciferase activities of WT and MUT RP11-297P16.4 following miR-145-5p mimic or miR-NC cotransfection and WT or MUT RP11-297P16.4 vector into 293T, A549, and H1299 cells (ns: not significant). (**E**) MiR-145-5p levels in H1299 and A549 cells after the silencing of RP11-297P16.4. Data are presented as mean ± SD; * *p* < 0.05, ** *p* < 0.01, and *** *p* < 0.001.

The subcellular localization of the lncRNA RP11-297P16.4 was assessed through a subcellular fractionation assay. RT-qPCR assay was subsequently conducted on cytoplasmic/nuclear RNAs to detect nuclear/cytoplasmic control transcript (U6/GAPDH separately) and RP11-297P16.4 expression. The results showed that it was predominantly localized in the cytoplasm, with a smaller fraction present in the nucleus (Figure 6A).

Using the human lncRNA target prediction tool in the miRDB database, we obtained multiple possible miRNA targets of lncRNAs. Notably, miR-145-5p, a miRNA that regulates cancer progression, was identified as a potential target [24,25]. The levels of the target miR-145-5p were measured in LUAD tissues in the TCGA dataset. The results of the Pearson correlation analysis showed that miR-145-5p was strongly negatively correlated to the lncRNA RP11-297P16.4 level (*r* = −0.56, *p* < 0.05; Figure 6B). The miR-145-5p binding sites for RP11-297P16.4-WT or RP11-297P16.4-MUT are shown in Figure 6C. To further identify whether miR-145-5p could bind to the predicted target sites in RP11-297P16.4, WT and MUT (putative binding sites for miR-145-5p were mutated) RP11-297P16.4 luciferase reporter vectors were constructed. As expected, co-transfection of the wild type RP11-297P16.4 vector (RP11-297P16.4-WT) with miR-145-5p mimics, but not the mutant RP11-297P16.4 vector (RP11-297P16.4-MUT), significantly reduced luciferase activities in 293T, A549 and H1299 cells (Figure 6D). Moreover, knocking down RP11-297P16.4 upregulated miR-145-5p in A549 and H1299 cells (Figure 6E). These findings indicated that RP11-297P16.4 is the ceRNA that binds to miR-145-5p.

### 3.5. MiR-145-5p Exerts an Antioncogenic Effect on NSCLC

**Figure 7:**
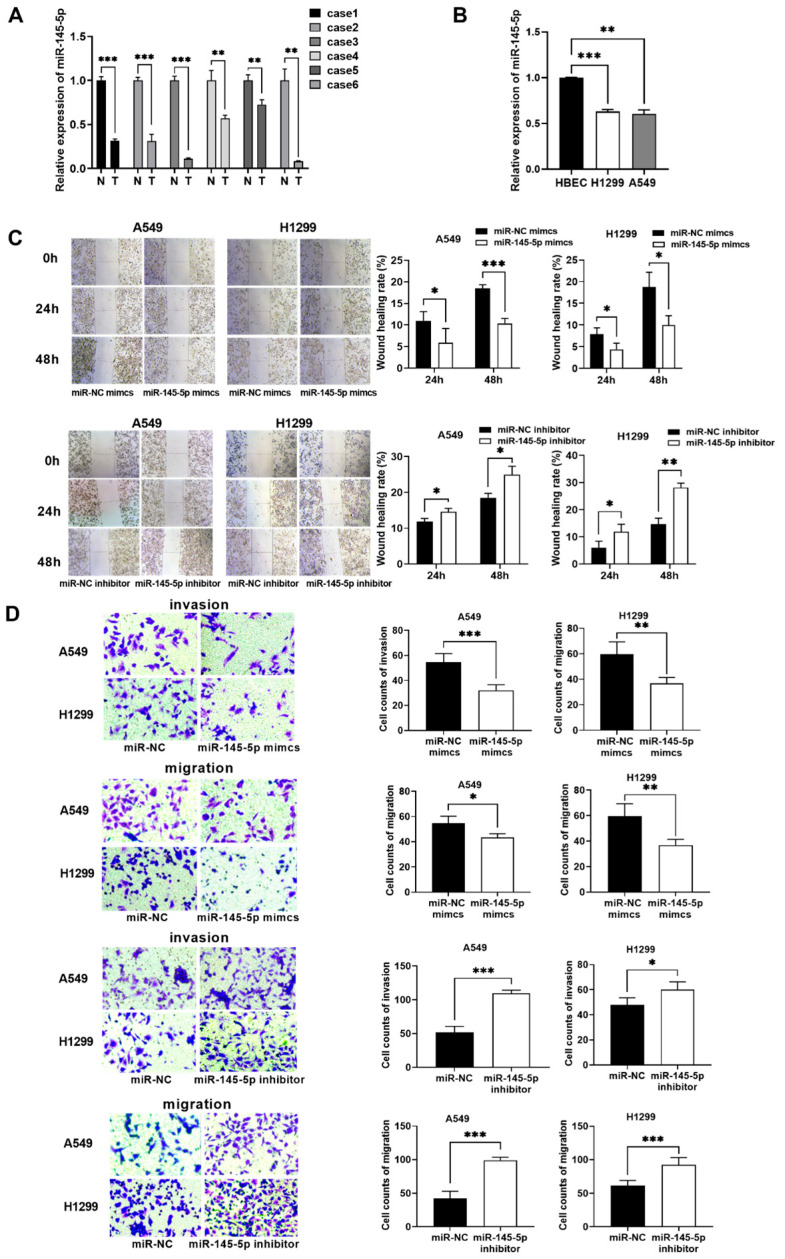
MiR-145-5p exerted an antioncogenic effect on NSCLC. (**A**) MiR-145-5p levels in six paired LUAD tissues and matched non-carcinoma samples were determined by RT-qPCR assays. (**B**) MiR-145-5p expression in A549 H1299 cells and HBECs was analyzed via RT-qPCR. (**C**) Metastatic ability of A549 and H1299 cells after miR-145-5p knockdown or overexpression was determined by conducting wound healing assays. (**D**) Metastatic ability of A549 and H1299 cells after miR-145-5p knockdown or overexpression was determined by conducting transwell assays. Data are presented as the mean ± SD; * *p* < 0.05, ** *p* < 0.01, and *** *p* < 0.001.

The miR-145-5p levels were considerably lower in LUAD samples than in non-carcinoma samples (Figure 7A). The miR-145-5p levels in NSCLC cells (A549 and H1299) and HBECs were determined by RT-qPCR assays, and their findings were similar to those of the tissue expression profiles (Figure 7B).

To elucidate the effect of lncRNA RP11-297P16.4-targeted miR-145-5p on NSCLC cell invasion and migration, miR-145-5p mimics and inhibitors (anti-miR-145-5p) were transfected into A549 and H1299 cells. In the wound healing assay, after the transfection of the miR-145-5p mimics, the healing of cells was slower than that recorded in the negative control (Figure 7C). In the transwell experiments, miR-145-5p overexpression decreased cell invasion and migration (Figure 7D). In contrast, treating these cancer cells with the miR-145-5p inhibitor promoted migration and invasion (Figure 7D).

### 3.6. Molecular Prediction of miR-145-5p Downstream mRNAs and Their Regulatory Relationships

**Figure 8:**
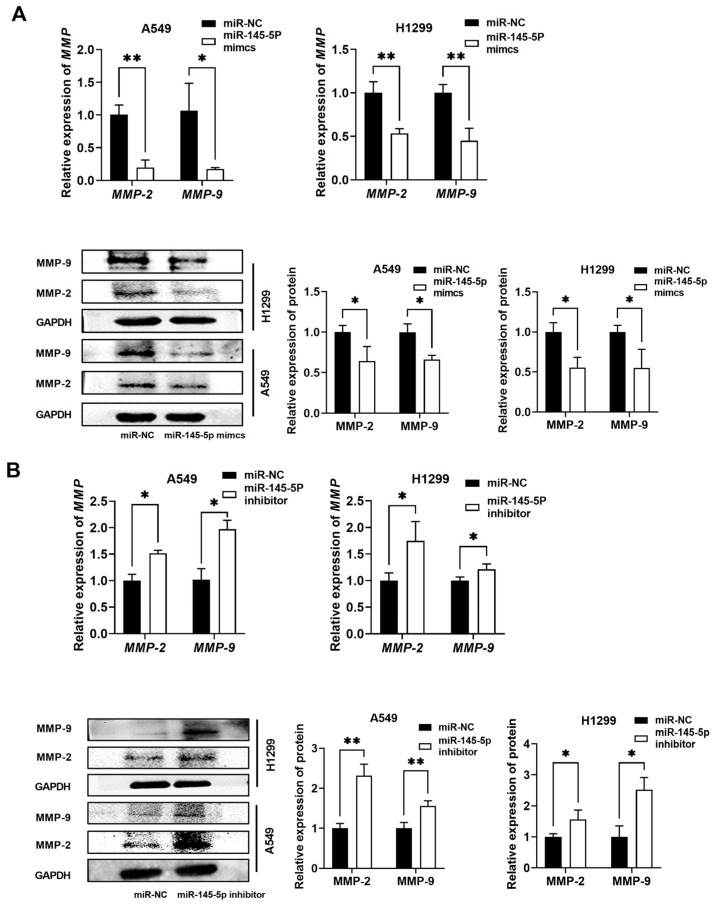
MiR-145-5p was negatively associated with MMP-2/9 levels in NSCLC. RT-qPCR and Western blot analysis of MMP-2/9 levels following miR-145-5p mimic (**A**) and miR-145-5p inhibitor (**B**) transfection in A549 and H1299 cells. Data are presented as mean ± SD; * *p* < 0.05, ** *p* < 0.01.

Bioinformatics databases and relevant literature reports were used to determine that miR-145-5p interacts directly and indirectly with MMP-2 and MMP-9 mRNAs, establishing a negative association between the expression of miR-145-5p and MMP-2/9 [26,27,28]. To validate the interaction of miR-145-5p with MMP-2 and MMP-9, we transfected A549 and H1299 cells with miR-145-5p mimics, miR-NC mimics, a miR-145-5p inhibitor, or a miR-NC inhibitor. The MMP-2 and MMP-9 gene mRNA expression and protein levels were subsequently analyzed via RT-qPCR and Western blot. Based on our findings, transfection with the miR-145-5p overexpression vector significantly inhibited MMP-2 and MMP-9 gene and protein expression in A549 and H1299 cells (Figure 8A). In contrast, miR-145-5p silencing upregulated MMP-2 and MMP-9 mRNA and protein levels, suggesting a key function of miR-145-5p in lung cancer cells through the regulation of MMP-2 and MMP-9 (Figure 8B).

### 3.7. LncRNA RP11-297P16.4 Targets miR-145-5p to Regulate MMP-2/9 Levels and Promote NSCLC Cells Invasion and Migration

**Figure 9:**
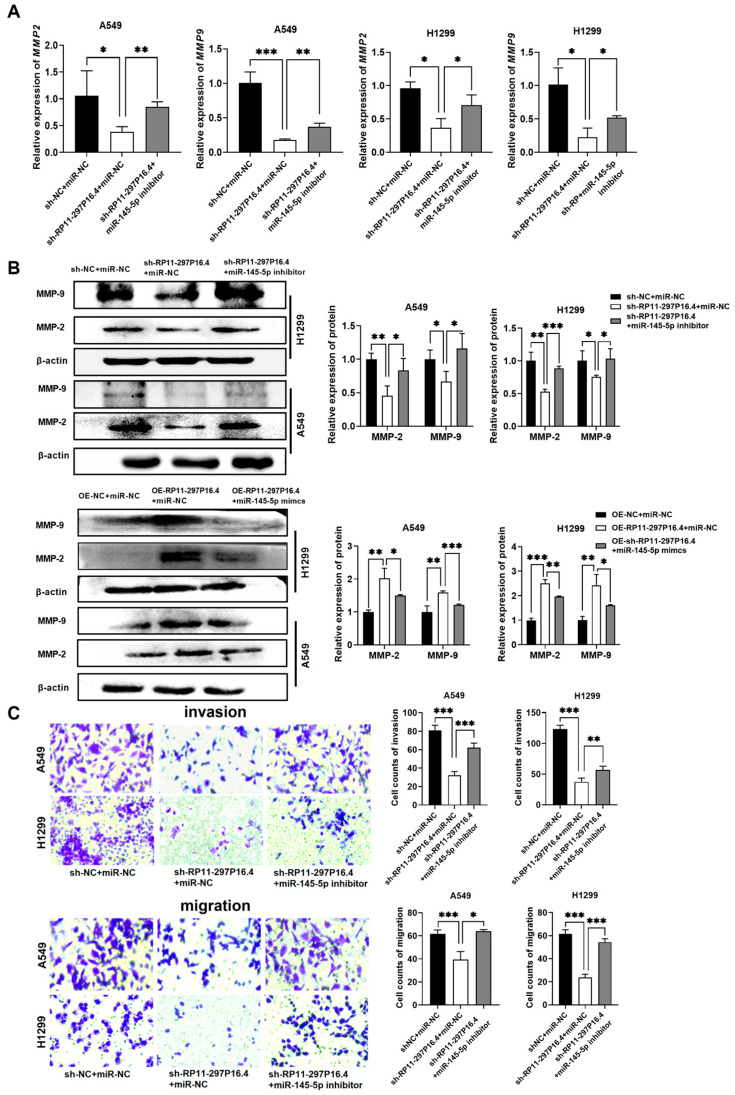
MiR-145-5p reversed lncRNA RP11-297P16.4-induced MMP-2/9 expression in NSCLC cells. (**A**) MMP-2/9 levels were detected by RT-qPCR after A549 and H1299 cells were co-transfected with sh-RP11-297P16.4 or the miR-145-5p inhibitor. (**B**) MMP-2/9 levels were detected by Western blot analysis following cotransfection with sh-RP11-297P16.4 and the miR-145-5p inhibitor or the RP11-297P16.4 plasmid (OE-RP11-297P16.4) and the miR-145-5p mimic into these cells. (**C**) Transwell assays were performed to assess NSCLC cell migration ability following treatment with sh-RP11-297P16.4 and the miR-145-5p inhibitor. Data are presented as mean ± SD; * *p* < 0.05, ** *p* < 0.01, and *** *p* < 0.001.

To better understand the interplay among lncRNA RP11-297P16.4, miR-145-5p, and MMP-2/9, we conducted a dual knockdown of lncRNA RP11-297P16.4 and miR-145-5p in NSCLC cells. Silencing of lncRNA RP11-297P16.4 decreased MMP-2 and MMP-9 mRNA levels in H1299 and A549 cells, whereas cotransfection with the miR-145-5p inhibitor upregulated their expression (Figure 9A). Moreover, MMP-2/9 protein levels decreased following lncRNA RP11-297P16.4 knockdown, while miR-145-5p silencing eliminated these effects (Figure 9B). Overexpression of RP11-297P16.4 increased the protein levels of MMP-2/9, while miR-145-5p mimic transfection eliminated these changes. (Figure 9B).

Concurrently, silencing of lncRNA RP11-297P16.4 suppressed the invasion and migration of NSCLC cells. LncRNA RP11-297P16.4 and miR-145-5p double knockdown promoted cell invasion and migration compared to lncRNA RP11-297P16.4 knockdown (Figure 9C). These findings suggested that the lncRNA RP11-297P16.4 influences the invasion and migration of A549 and H1299 cells by regulating miR-145-5p and MMP-2/9.

## 4. Discussion

The factors that drive the occurrence of NSCLC include genetic changes (such as mutated oncogenes, oncogenic alterations, and loss of tumor suppressor genes), sex, habits and customs (such as smoking), and environmental hazards (such as air pollutants) [29]. Investigating the expression patterns and functions of potential oncogenes that are highly expressed in NSCLC is important to elucidate the mechanism underlying NSCLC and identify therapeutic targets.

Long noncoding RNAs (lncRNAs), which are longer than 200 nucleotides, have recently received considerable attention as key players in various biological processes [30]. Based on their genomic location, lncRNAs are grouped into intergenic, intronic, divergent, and antisense types [31]. More than 23,000 lncRNAs are present in the human genome, many of which have important functions in normal and cancerous cellular processes [32]. The mechanisms by which lncRNAs participate in various disease processes are not clear and need to be investigated. For example, the lncRNA metastasis-associated lung adenocarcinoma transcript 1 (MALAT1) overexpression induces reprogramming of the tumor microenvironment in LUAD to drive metastasis [33]. In ovarian tumor, the inhibition of MALAT1 has significantly impeded EMT-related genes [34]. In hepatocellular carcinoma, overexpressed lncRNA MVIH was significantly associated with frequent microvascular invasion, higher tumor node metastasis stage, and overall survival [35]. In this study, the expression patterns of the lncRNA RP11-297P16.4 in LUAD patients and NSCLC cell lines were measured, and the results revealed significant upregulation of the expression of the lncRNA RP11-297P16.4 in tumor tissues and LUAD cell lines. Next, the effects of RP11-297P16.4 on NSCLC cell lines were investigated, and the results showed that RP11-297P16.4 promoted cell invasion and migration but not cell proliferation.

Matrix metalloproteinases (MMPs) were historically described as essential facilitators of tumor metastasis [36] and responsible for decomposing the BM and ECM [37]. Tumor cells invaded the surrounding tissue based on the ability of degrading the BM and ECM [38]. Among MMPs, the overexpression of MMP-2 and MMP-9 and their increased activities are closely related to BM and ECM decomposition, which increased tumor cell invasion and migration ability [39,40]. Experimental results had substantiated that lncRNA RP11-297P16.4 promoted the expression of MMP-2 and MMP-9 in NSCLC cells, and these results explained why RP11-297P16.4 promoted the invasion and migration of NSCLC cells.

LncRNAs distributed in different parts of the cell exert their effects through different mechanisms. For example, in the nucleus, they function by directing the transcriptional complex or chromatin assembly to regulate the transcription and epigenetic modification of the genome [41,42]. In the cytoplasm, lncRNAs function via allosteric regulation, modulation of cell signaling, post-transcriptional mRNA processing, and post-translational modifications [43,44]. LncRNAs can act as ceRNAs to regulate the expression of target mRNAs via the combination of shared miRNAs [45]. In this study, the lncRNA RP11-297P16.4 was found to be distributed in the cytoplasm, suggesting that the lncRNA—miRNA-mRNA ceRNA network may be involved in the function of the lncRNA RP11-297P16.4 in NSCLC cells. Bioinformatics analysis combined with a dual luciferase reporter assay indicated that miR-145-5p was the target miRNA. MiR-145-5p regulates MMP-2 and MMP-9 mRNAs directly [26] or indirectly [46,47]. There are, however, several limitations. While rodent models are widely used in preclinical research, their inherent physiological differences from humans may fail to fully recapitulate the natural progression, heterogeneity, and microenvironment of human tumors. Additionally, these researchers only performed dual-luciferase reporter assays to verify that MMP-2 is a direct target of miR-145-5p. And all these studies did not involve the clinical characteristics, such as tumor-node-metastasis (TNM) stage of patients with LUAD. Further experimental study is needed to verify the potential biological mechanisms of RP11-297P16.4/miR-145-5p/MMP-2/9 axis in NSCLC.

## 5. Conclusions

To summarize, it was found that RP11-297P16.4 is upregulated in NSCLC. The knockdown of RP11-297P16.4 inhibits invasion and metastasis in NSCLC cell lines. RP11-297P16.4 functions as a ceRNA to regulate MMP-2/9 expression by decoying miR-145-5p. CeRNA networks offers critical insights for precision oncology, as RNA molecules are more vulnerable to intervention than proteins. By developing tumor-selective approaches to modulate key regulatory elements, including oncogenic lncRNAs, tumor-suppressive miRNAs, and metastasis-associated mRNAs, we can revolutionize therapeutic development while reducing the off-target toxicity characteristic of conventional treatments.

## Data Availability

All of the primary data and materials involved in this paper are from the primary data source. If readers need more information about data and materials, please contact the author for data requests.

## Abbreviations

The following abbreviations are used in this manuscript:

lncRNAs	long noncoding RNAs
ceRNA	competitive endogenous RNA
MMP	matrix metalloproteinase
LUAD	lung adenocarcinoma
NSCLC	non-small-cell lung carcinoma
